# A Randomized, Observer-Blinded, Active-Controlled, Phase IIIb Study to Compare IV/Oral Delafloxacin Fixed-Dose Monotherapy With Best Available Treatments in a Microbiologically Enriched Population With Surgical Site Infections: The DRESS Study

**DOI:** 10.1093/ofid/ofaf476

**Published:** 2025-08-29

**Authors:** Nikolay Belev, Andres Tein, Giuseppe Mangialardi, Alessandra Nuti, Giovanni Marino Merlo, Simona Scartoni, Monica Bertolotti, Margherita Lerro, Stefano Margaritora

**Affiliations:** University Multiprofile Hospital for Active Treatment “Eurohospital Plovdiv,” Plovdiv, Bulgaria; Department of Surgery, Tartu University Hospital, Tartu, Estonia; Global Clinical Sciences, Menarini Ricerche SpA, Florence, Italy; Global Clinical Sciences, Menarini Ricerche SpA, Florence, Italy; Global Clinical Sciences, Menarini Ricerche SpA, Florence, Italy; Global Clinical Sciences, Menarini Ricerche SpA, Florence, Italy; Global Clinical Sciences, Menarini Ricerche SpA, Florence, Italy; Global Clinical Sciences, Menarini Ricerche SpA, Florence, Italy; Faculty of Medicine and Surgery, Catholic University of Sacred Heart, Rome, Italy; Thoracic Surgery Unit, University Hospital Foundation Agostino Gemelli IRCCS, Rome, Italy

**Keywords:** acute bacterial skin and skin structure infections (ABSSSIs), clinical success, delafloxacin, safety, surgical site infections (SSIs)

## Abstract

**Background:**

Surgical site infections (SSIs) are the most common skin and skin structure infections and are mostly polymicrobial, requiring hospitalization and broad-spectrum antibiotics. This clinical trial evaluated the noninferiority of delafloxacin vs best available therapy (BAT) for the treatment of superficial or deep incisional SSI following a cardiothoracic/related leg or abdominal surgical procedure.

**Methods:**

In this randomized, observer-blinded, active-controlled, parallel-group, multicenter, phase IIIb study, patients with SSI were randomized 1:1 to receive delafloxacin 300 mg intravenous (IV)/450 mg oral (OS) or BAT IV/OS (vancomycin or linezolid for cardiothoracic SSI, piperacillin/tazobactam or tigecycline for abdominal SSI). The primary end point was clinical success, defined as the clinical response (cure or improved) at test of cure (TOC), performed 7–14 days after end of treatment (EOT) visit. Secondary end points were clinical success at EOT, sustained clinical response at last follow-up (LFU), microbiological response, and safety.

**Results:**

Thi study enrolled 266 patients (delafloxacin = 134; BAT = 132) with comparable baseline characteristics between the 2 treatment arms. Delafloxacin clinical success was noninferior vs BAT at TOC visit (91.8% vs 90.2%, respectively). Similar efficacy was confirmed at LFU (91.8% delafloxacin; 87.9% BAT). Comparable microbiological response was obtained with delafloxacin (89.5%) and BAT (79.4%). Delafloxacin and BAT demonstrated comparable treatment adverse event rates (23.9% and 19.7%, respectively), mostly mild-to-moderate gastrointestinal reactions.

**Conclusions:**

This study provided new data on delafloxacin in SSIs, covering the need for effective empiric treatment against the wide spectrum of pathogens involved in these infections.

**Clinical Trials registration:**

NCT04042077; 2018-001082-17 (EudraCT).

Acute bacterial skin and skin structure infections (ABSSSIs) encompass a variety of disease presentations and severities involving the skin and underlying subcutaneous tissue, fascia, or muscle and ranging from simple superficial infections to severe necrotizing infections [1, 2].

ABSSSIs are often polymicrobial, including both aerobe and anaerobe, gram-positive (staphylococci, enterococci) and gram-negative bacteria (*Enterobacterales*, *Pseudomonas aeruginosa*), with gram-negative pathogens more frequent in SSIs occurring after abdominal surgery, thus requiring broad-spectrum antibacterials [1, 3–5].

The increase in hospital admissions required to treat ABSSSIs with intravenous (IV) antibiotics, along with the spread of multidrug-resistant (MDR) bacteria, has caused a considerable impact on hospital stays and patient morbidity, reinforcing the need for new treatment options [6].

Vancomycin IV and linezolid IV or oral formulations are most often specifically recommended for infections caused by MRSA and only inhibit gram-positive bacteria [7, 8].

Linezolid is also associated with high cost and with toxicity in case of long treatment duration or in some at-risk patients [9]. On the other hand, vancomycin, due to the decreased susceptibility against MRSA strains, is recommended at the higher range of therapeutic doses, which can result in renal toxicity [10].

Both tigecycline and piperacillin-tazobactam show broad-spectrum activity. However, tigecycline is intended for IV use only with twice-daily administration, while piperacillin-tazobactam IV is usually given in divided doses every 6 to 8 hours. Moreover, tigecycline requires hospitalization in most cases, and frequent nausea is a common adverse event [11].

New-generation antibiotics introduced the chance to modulate clinical management based on patient comorbidity, expected adherence to outpatient therapy, early discharge or avoiding hospitalization with oral formulations, early switch from intravenous to oral therapy, or single-dose administration of long-acting intravenous agents [12]. Delafloxacin is a new fluoroquinolone antibiotic, approved in 2019 for treatment of ABSSSIs caused by both gram-positive and gram-negative organisms. Delafloxacin dually targets and equally inhibits the essential bacterial enzymes topoisomerase IV and DNA gyrase (topoisomerase II), while other fluoroquinolones show more potent inhibition for only 1 of these at time, exhibiting a broad spectrum of activity against clinically relevant microorganisms and low risk of the emergence of resistance among susceptible pathogens [3, 13]. Its anionic structure enhances its potency in acidic environments, which are typical of sites of infection, including skin and soft tissue infections caused by *S. aureus* [14]. Delafloxacin has shown greater in vitro potency (at least 64 times) against staphylococci including MRSA and is at least 4 times more potent against *P. aeruginosa* when compared with other fluoroquinolones such as ciprofloxacin and levofloxacin in patients with ABSSSI and osteomyelitis [15]. In a resistance selection study, delafloxacin appeared to have greater stability against target enzyme mutations in gram-positive bacteria compared with other fluoroquinolones such as ciprofloxacin, levofloxacin, and moxifloxacin in MRSA isolates [16].

Delafloxacin also shows high activity against anaerobes, with low minimum inhibitory concentrations (MICs) for *Clostridium* spp., *Prevotella* spp., and *Bacteroides fragilis* against other gram-positive anaerobic rods (ie, *Cutibacterium* acnes, *Propionibacterium avidum*, *Actinomyces* spp.) and mycobacteria including *Legionella pneumophila* and *M. pneumoniae* [13].

The main goal of the Delafloxacin intRavenous and oral monotherapy in Surgical Site infections (DRESS) study was to assess the efficacy and safety of delafloxacin compared with best available therapy (BAT), selected among the current standard of care, in the treatment of superficial or deep leg surgical site infections (SSIs), which are a known complication following cardiothoracic procedures, or abdominal SSI.

## METHODS

### Study Population

The DRESS study was a randomized, observer-blinded, active-controlled, parallel-group, multicenter, phase IIIb trial evaluating the efficacy and safety of delafloxacin compared with selected BATs for the treatment of superficial or deep SSI following cardiothoracic surgery or leg surgery for coronary artery bypass grafting (CABG; abbreviated with “cardiothoracic/related leg” in the text) and abdominal SSI (ie, an expected microbiologically enriched SSI).

Patients were enrolled at 66 centers from 14 European countries (Austria, Bulgaria, Croatia, Czech Republic, Estonia, Hungary, Italy, Latvia, Poland, Romania, Serbia, Slovenia, Spain, and the UK) between September 2019 and October 2020. A sample size of 600 randomized patients (300 patients for each treatment arm) was planned to provide 80% power in the intent-to-treat (ITT) population and >95% in the clinically evaluable (CE) population to demonstrate the noninferiority of delafloxacin vs reference treatment arms in terms of clinical response (clinical success) rate with a noninferiority margin of 10% and an alpha equal to .025 (1-side test).

### Eligibility Criteria

Patients included in the study were adult males or females (age >18 years) with a history of cardiothoracic/related leg or abdominal surgery occurring within the past 30 days and no implant left in place, a diagnosis of superficial or deep incisional SSI, and an infection severity requiring IV treatment and patient hospitalization according to the investigator's judgment.

Superficial or deep incisional SSIs were diagnosed according to the Centers for Disease Control and Prevention (CDC) definition [2]. Superficial incisional SSI involves only the skin and subcutaneous tissue of the incision and at least 1 of the following local findings: purulent drainage from the superficial incision; organisms isolated from an aseptically obtained culture of fluid or tissue from the superficial incision; a superficial incision deliberately explored by a surgeon AND the patient having at least 1 of the following signs or symptoms of infection (pain or tenderness, localized swelling, redness or heat); diagnosis of superficial incisional SSI made by the investigator. Deep incisional SSI involved deep soft tissues (eg, fascia and muscle layers) at the incision site and at least 1 of the following findings: purulent drainage from the deep incision but not from the organ/space component of the surgical site; a deep incision spontaneously dehiscing or being deliberately opened by a surgeon with the patient having at least 1 of the following signs or symptoms of infection (fever >38°C, localized pain or tenderness); an abscess or other evidence of infection involving the deep incision found on direct examination, during reoperation, or by histopathologic or radiologic examination; diagnosis of deep incisional SSI made by the investigator. Patients were excluded from the study if they had 1 of the criteria listed in Table 1.

**Table 1.** Key Exclusion Criteria

Treatment with an antimicrobial therapy >24 h and 72 h before the first dose of study treatment Implant left in place after cardiothoracic/related leg or abdominal surgery Any infection requiring treatment with systemic antimicrobial agents other than the ones included in the study Medical history of significant hypersensitivity/allergic reaction/contraindication to the study drugs or drug excipients Underlying medical conditions (eg, severe cerebral arteriosclerosis, epilepsy, myasthenia gravis, diarrhea due to *C. difficile*, complicated intra-abdominal infection) Any chronic or underlying skin pathology at the site of infection with the potential to complicate the assessment of clinical response (eg, atopic dermatitis or eczema) or any skin condition that, in the opinion of the investigator, might interfere with SSI healing Deep immunosuppressive status Treatment with systemic corticosteroids for >10 d at a dose equivalent to >15 mg of prednisolone in the previous 2 wk Patients with end-stage renal disease on hemodialysis or peritoneal dialysis or creatinine clearance <15 mL/min using the Cockcroft-Gault formula

Abbreviation: SSI, surgical site infection.

### Outcomes

The primary efficacy end point was clinical success, defined as the clinical response of “cure” or “improved” at test of cure (TOC) 7–14 days after the last dose in the ITT and CE populations of patients with superficial or deep incisional SSI, following a cardiothoracic/related leg or abdominal surgical procedure.

Additional efficacy analyses were performed to assess clinical success and microbiological responses at different study time points. Additionally, hospital infection-related length of stay (IRLOS), hospital length of stay (LOS), and duration of overall treatment IV and OS were analyzed. During the study, safety and tolerability data were also collected to provide additional information on the profile of delafloxacin.

SSI microbiological assessment was based on microbiological cultures from the primary infection site and blood sample results, and microbiological isolates were identified by validated culture methods. Antimicrobial susceptibility tests were performed according to Clinical and Laboratory Standards Institute (CLSI) and European Committee on Antimicrobial Susceptibility Testing (EUCAST) guidelines for fastidious organisms [17, 18].

The lowest minimum inhibitory concentration (MIC) inhibiting 50% or 90% of the strains within a single species (MIC50 or MIC90, respectively) was calculated for the most common pathogens considering all tests performed at all visits.

**Figure 1.**
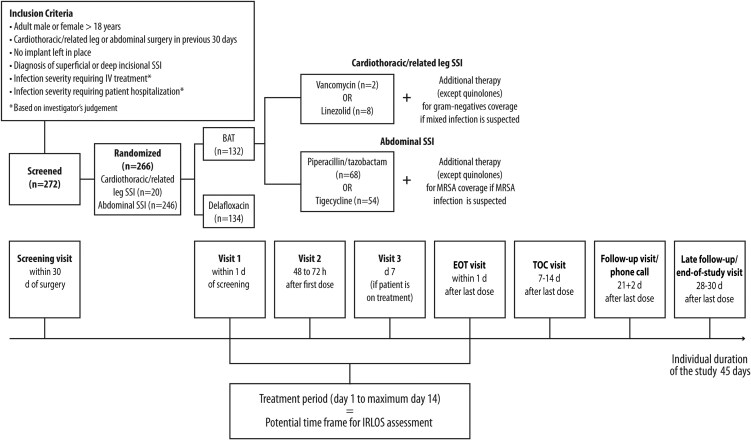
Visits and treatment schedule of the DRESS study. Abbreviations: BAT, best available therapy; DRESS, Delafloxacin intRavenous and oral monothErapy in Surgical Site infections; IRLOS, infection-related length of stay; SSI, surgical site infection.

The study encompassed up to 8 site visits ending with the LFU visit, which represented the end of study visit. The study duration was determined individually for each patient, based on duration of study treatment (5- to 14-day interval), with an overall individual study duration of at most 45 days (Figure 1).

Patients were considered eligible to be discharged by the blinded observer.

### Study Treatment

Eligible patients were randomly assigned 1:1 to receive delafloxacin or BAT, selected between 2 options for each SSI based on the investigator's judgment given the patient's characteristics and local epidemiological pattern. The BAT was selected to address the microbiological flora causative of postcardiothoracic and abdominal SSI as described below, based on international guidelines [19, 20].

Delafloxacin 300 mg IV was given every 12 hours, with the option to switch to delafloxacin 450 mg oral (OS) every 12 hours as soon as the patient met the eligibility criteria to switch to an oral formulation, as per a blinded observer's assessment. Delafloxacin infusions lasted 60 minutes. BAT for patients with cardiothoracic/related leg SSI was vancomycin 15 mg/kg given IV every 12 hours or linezolid 600 mg IV every 12 hours with the option to switch to linezolid 600 mg OS every 12 hours as soon as the patient met the eligibility criteria to switch to an oral formulation, as per a blinded observer's assessment. The BAT for abdominal SSI was piperacillin/tazobactam 4 g/0.5 g IV given every 8 hours or a tigecycline 100 mg IV loading dose, followed by 50 mg IV given every 12 hours. The selection of 1 of the 2 BAT options for each SSI was based on the investigator's judgment given the patient’s characteristics and the local epidemiological pattern. In patients with renal impairment, the dosing of renally cleared drugs was adjusted based on creatinine clearance. Additional therapy could also be prescribed by the investigator to optimize the microbiological activity of BAT if mixed gram-negative infection in cardiothoracic SSI patients or methicillin-resistant *Staphylococcus aureus* (MRSA) in abdominal SSI patients (in the Piperacillin/Tazobactam treatment group) was suspected, indicated as per local standard of care and with the only exclusion being quinolones.

Treatment duration of the study was based on the results of delafloxacin pivotal trials and according to the SmPCs of the reference treatments [9] [21–23], ranging from minimum 5 to maximum 14 days, with enrolled patients’ exposure to the study drug according to the investigator’s judgment.

### Data Collection

Patients were stratified by site of infection (either cardiothoracic/related leg SSI or abdominal SSI) and by superficial or deep infections.

At each visit, the investigator assessed the signs and symptoms of SSI as anatomical site of infection and classification of superficial or deep SSI (at screening only), as well as presence/absence of drainage/discharge, fluctuance, heat/localized warmth, swelling/induration, pain/tenderness, erythema/extension of redness (and maximum extension from the wound edge in mm), and lymphangitis and lymphadenopathy (with number and anatomical site of lymph nodes).

SSI clinical response was based on patient assessment of infection signs and symptoms at the EOT, TOC, and late follow-up visits and classified as cure (complete resolution of all baseline signs and symptoms of SSI), improved (resolution of 2 or more signs and/or symptoms, but not all, with an improvement to such an extent that no additional antibiotic treatment was necessary), failure (any administration of antibacterial therapy for SSI because of lack of efficacy of the study treatment after 2 days, need to continue study treatment >14 days, need for surgical intervention, or need for antibiotic therapy for *P. aeruginosa* only for tigecycline-treated patients), or missing or indeterminate (not assigned because of incomplete assessments or because the patient received potentially effective nonstudy antibacterial drug therapy for the treatment of a condition other than SSI).

Clinical success included the response of cure or improved. A clinical response of failure at a visit was considered failure at any subsequent visit. A missing response was considered failure for the analyses on the ITT population, and the patient was to be excluded from the computably enumerable (CE) population. The CE population refers to all subjects in the ITT population who met the following criteria: (1) diagnosis of cardiothoracic/related leg or abdominal SSI; received the correct treatment based on the randomization assignment; (2) received 80% of the expected doses of the study drug in the treatment period; (3) did not receive any concomitant, systemic antibacterial therapy except for lack of efficacy; (4) had no protocol deviations that would affect assessment of efficacy at the reference visit. CE populations were defined at the EOT, TOC, and LFU time points and for eligibility to switch to oral formulation, IRLOS, and LOS.

An indeterminate response was considered failure for the analyses on the ITT population, and the patient was excluded from the CE population.

The bacterial pathogens identified from the baseline blood culture and the bacterial pathogens from the SSI were listed separately. The blinded external expert reviewed and identified which organisms were causative pathogens of SSI and assigned the correspondent microbiological response for each causative pathogen among the following definitions: documented eradicated (baseline pathogen absent in cultures from EOT and TOC visits), documented persisted (baseline pathogen present in cultures from EOT and TOC visits), not evaluable (not feasible to assess the microbiological response/no material available for specimen), and new pathogen (the present pathogen is known to cause SSI and differs from the pathogen detected at baseline). If not evaluable, microbiological response was defined based on clinical response at the EOT and TOC visits as “presumed” eradicated (with clinical response of “success”), presumed persisted (with clinical response of “failure”), or presumed indeterminate (with “indeterminate” clinical response).

When microbiological samples taken postbaseline through the TOC from the original site of infection were positive for “new” pathogen(s), these infections were classified as emergent and defined as superinfections or new infections if the new pathogen known to cause SSI was cultured during treatment or after the end of treatment with a clinical response of “failure.”

AEs were coded using the MedDRA dictionary—version 23.0—and summarized by treatment arm/individual treatment. Treatment-emergent adverse event (TEAE) was defined as any AE that starts after the first dose of study drug or worsens in intensity after the first dose of study drug through follow-up. Any TEAE was defined as a related adverse event (ADR) if the causality category fell into 1 of the following: certainly related, probably related, possibly related, and nonassessable/unclassifiable. Patients with 1 or more reported events were counted only once.

### Statistical Analysis

The primary efficacy variable, “clinical success,” defined as “cure” or “improved” at TOC, was used to assess the noninferiority of delafloxacin vs the reference treatment arm in the ITT and CE populations. The treatment difference in response rate (delafloxacin minus the reference treatment arm) was presented with the relative 2-sided 95% CI. With a lower limit >−0.10, delafloxacin was concluded to be noninferior to the reference treatment arm for treating patients with cardiothoracic/related leg or abdominal SSI. If noninferiority was confirmed, the superiority of delafloxacin vs the reference treatment arm was to be tested on the ITT population by a chi^2^ test.

All the secondary efficacy end points were descriptively analyzed and tested, when applicable, for noninferiority with the possibility to switch to superiority through an ad hoc inferential analysis: Binary outcomes were analyzed as for the primary efficacy end point; continuous variables were tested by using an analysis of variance model with treatment arm as the main factor; time to event variables were assessed using a log-rank test.

Safety variables were analyzed only by descriptive statistics and were run on the safety population.

## RESULTS

### Overall Cohort

Due to the significant impact of the coronavirus disease 2019 (COVID-19) pandemic, the study ended early. The number of patients for each data set analyzed is reported in Table 2. At that time point, a total of 266 patients, most of whom were in the abdominal SSI setting, were included in the ITT population. Patients were females and males with a mean age of 64.9 years. Of these, 92.5% of randomized patients suffered from an abdominal SSI (n = 246), while only 7.5% had a cardiothoracic/leg-related SSI (n = 20). Superficial infections were the most present SSIs (60.5%) in comparison with deep ones (39.5%). No significant differences in demographics or other baseline characteristics emerged between the delafloxacin and BAT groups (Table 3).

**Table 2.** Patients’ Disposition by Analysis Population

Populations		Delafloxacin	BAT
Safety population		134	132
ITT population		134	132
MITT population		105	102
CE population	EOT	120	126
	TOC	116	119
	LFU	114	116
	Potential switch	128	127
	IRLOS	128	127
	LOS	130	127
ME population	EOT	95	98
	TOC	91	93

ITT population: all randomized and treated subjects analyzed according to the randomized treatment arm (test or reference). MITT population: all subjects in the ITT population who had bacterial pathogens identified at baseline that were known to cause cardiothoracic/related leg and/or abdominal SSI. CE population: all subjects in the ITT population who met the following criteria: (1) diagnosis of cardiothoracic/related leg or abdominal SSI; received the correct treatment based on the randomization assignment; (2) received 80% of the expected doses of study drug in the treatment period; (3) did not receive any concomitant, systemic antibacterial therapy except for lack of efficacy; (4) had no protocol deviations that would affect assessment of efficacy at the reference visit. CE populations were defined at the EOT, TOC, and LFU time points and for eligibility to switch to oral formulation, IRLOS, and LOS. ME population: all subjects in the MITT population who also met the criteria for the CE population. ME populations were defined at EOT and TOC. Safety population: all subjects who received at least 1 dose of study medication.

Abbreviations: BAT, best available therapy; CE, computably enumerable; EOT, end of treatment; IRLOS, infection-related length of stay; ITT, intention-to-treat; LFU, loss to follow-up; ME, microbiologically evaluable; MITT, modified intention-to-treat; SSI, surgical site infection; TOC, test of cure.

**Table 3.** Baseline Characteristics of the ITT Population

Variable	Delafloxacin (n = 134)	BAT (n = 132)	Overall (n = 266)
Age, mean (SD), y	66.0 (13.6)	63.7 (13.7)	64.9 (13.7)
Gender, No. (%)			
Female	61 (45.5)	53 (40.2)	114 (42.9)
Male	73 (54.5)	79 (59.8)	152 (57.1)
Race/ethnicity, No. (%)			
Hispanic or Latino	3 (2.2)	1 (0.8)	4 (1.5)
Not Hispanic or Latino	131 (97.8)	131 (99.2)	262 (98.5)
White	133 (99,3)	132 (100)	265 (99,6)
Black or African American	1 (0.7)	0	1 (0.4)
ABSSSI type, No. (%)			
Cardiothoracic/related leg SSI	10 (7.5)	10 (7.6)	20 (7.5)
Abdominal SSI	124 (92.5)	122 (92.4)	246 (92.5)
SSI type, No. (%)			
Superficial	82 (61.2)	79 (59.8)	161 (60.5)
Deep	52 (38.8)	53 (40.2)	105 (39.5)
Temperature, mean (SD),^a^ °C	37.2 (0.70)	37.2 (0.70)	37.2 (0.70)
Preexisting medical conditions, No. (%)			
Diabetes mellitus	17 (12,7)	12 (9.1)	29 (10.9)
Creatinine clearance, mean (SD),^a,b^ mL/min	99.7 (39.69)	101.7 (42.46)	100.6 (41.02)
Neuropathy complication	3 (2.2)	1 (0,7)	4 (1.5)
Benign/malign or unspecific neoplasm^c^	49 (36.6)	42 (31.8)	91 (34.2)
Obesity (BMI ≥30 kg/m^2^)	7 (5.2)	10 (7.6)	17 (6.4)
Chronic kidney disease	4 (3.0)	2 (1.5)	6 (2.2)
White blood cells, mean (SD),^a^ cells × 10^9^/L			
Basophils	0.05 (0.07)	0.04 (0.04)	0.05 (0.06)
Eosinophils	0.21 (0.24)	0.18 (0.18)	0.20 (0.21)
Leukocytes	9.97 (4.32)	10.03 (4.58)	10.00 (4.41)
Lymphocytes	1.66 (1.01)	1.52 (0.68)	1.59 (0.86)
Monocytes	0.80 (0.82)	0.73 (0.36)	0.76 (0.64)
Neutrophils	7.25 (3.80)	7.46 (4.17)	7.35 (3.98)
Prior medications, No. (%)^d^			
Anti-infectives for systemic use	73 (54.5)	67 (50.7)	140 (52.6)
Antineoplastic/immunomodulating agent	1 (0.7)	1 (0.7)	2 (0.7)
Antibiotics/chemotherapeutics for dermatological use	0	1 (0.8)	1 (0.4)
Antiseptic and disinfectants	1 (0.7)	1 (0.8)	2 (0.7)
Systemic corticosteroids^e^	28 (20.9)	26 (19.7)	54 (20.3)
Antinflammatory/antirheumatic drug	45 (33.6)	40 (30.3)	85 (31.9)

Abbreviations: ABSSSI, acute bacterial skin and skin structure infection; BAT, best available therapy; BMI, body mass index; ITT, intention-to-treat; SSI, surgical site infection.

^a^Labs data percentages calculated from the number of subjects in the respective group considering the safety population, n/Ni * 100%, where n is the number of subjects in each population and Ni is the numerosity of the i-th group (ie, number of subjects taking the i-th study treatment).

^b^Creatinine clearance <15 mL/min was calculated using the Cockcroft-Gault formula.

^c^Including cysts and polyps.

^d^Prior medication is a medication ended within 4 weeks of the screening date. Prior medications are coded using WHODrug-202003. The total number of medications counts all the medications for patients. At each level of patient summarization, a patient is counted once if the patient reported 1 or more medications: number of medications/number of subjects (percentage).

^e^Patients with a history of systemic corticosteroid use for >10 days at a dose equivalent to >15 mg prednisolone in the previous 2 weeks were excluded.

The mean treatment duration for patients receiving delafloxacin IV/OS was 7.5 days, whereas in the BAT group the mean duration of treatment was 6.7 days (5.8 days linezolid, 7.6 days piperacillin-tazobactam, 5.6 days tigecycline, and 9.1 days vancomycin) in the ITT population. Eighteen patients (9 in the delafloxacin group and 9 in the BAT group) discontinued the treatment due to adverse events (2 vs 2), lack of efficacy (2 vs 3), need for major surgical procedure (1 in the delafloxacin group), and investigator's decision (2 patients under BAT).

### Clinical Efficacy in SSI Treatment

Delafloxacin noninferiority compared with BAT was confirmed in both the CE and ITT populations. Clinical success at TOC was reported in 97.4% and 91.8% in the CE and ITT populations treated with delafloxacin, and 95% and 90.2% in the CE and ITT populations treated with BAT, respectively (ITT: common risk difference, 0.0171; 95% CI, −0.0568 to 0.0910; CE: common risk difference, 0.0322; 95% CI, −0.0325 to 0.0969).

**Figure 2.**
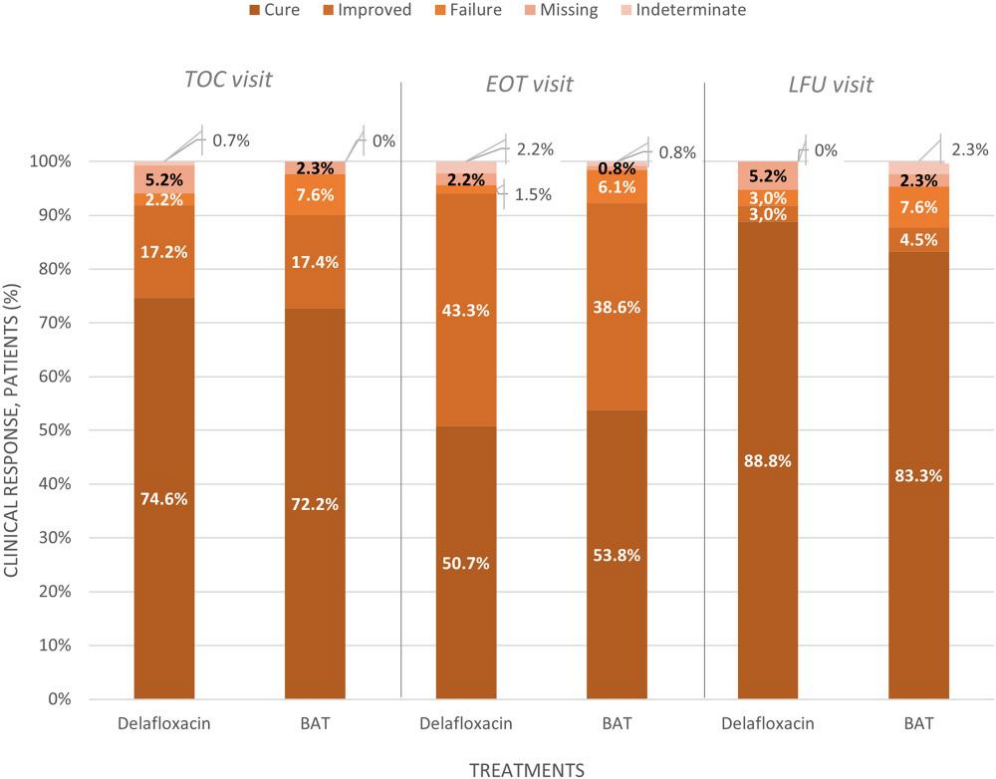
Clinical response at the TOC, EOT, and LFU time points in the ITT populations. At TOC visit: CRD = 0.0171 (95% CI, −0.0568 to 0.0910; *P* = .6661). Noninferiority: met. At EOT visit: CRD = 0.0001 (95% CI, −0.0660 to 0.0662; *P* = .6254). Noninferiority: met. At LFU visit: CRD = 0.0387 (95% CI, −0.0391 to 0.1165; *P* = .3126). Noninferiority: met. Confidence intervals were calculated using the Miettinen and Nurminen methods. If the LL of the 2-sided 95% CI was >−0.10, it was concluded that delafloxacin was noninferior to the reference treatment arm for treating patients with cardiothoracic or abdominal SSI. If the LL of the 2-sided 95% CI was >0, then delafloxacin was declared superior to the reference treatment arm for treating patients with cardiothoracic or abdominal SSI. The *P* value was calculated using the Mantel-Haenszel method. If the *P* value was lower than .05, then delafloxacin was declared superior to the reference treatment arm for treating patients with cardiothoracic or abdominal SSI. Abbreviations: BAT, best available therapy; CRD, common risk denominator; EOT, end of treatment; ITT, intention-to-treat; LFU, last follow-up; SSI, surgical site infection; TOC, test of cure.

Clinical success was reached with a similar distribution between the “cure” and “improved” response in both groups (CE population): 81% and 16.4% of patients treated with delafloxacin and 75.6% and 19.3% of patients treated with BAT, respectively (Figure 2). The superiority test did not achieve statistical significance (*P* = .6661).

At EOT visit, delafloxacin achieved 94% of clinical “success” compared with 92.4% of the BAT group in the ITT population (common risk difference, 0.0001; 95% CI, −0.0660 to 0.0662); no statistically significant superiority was demonstrated (*P* = .6254).

**Figure 3.**
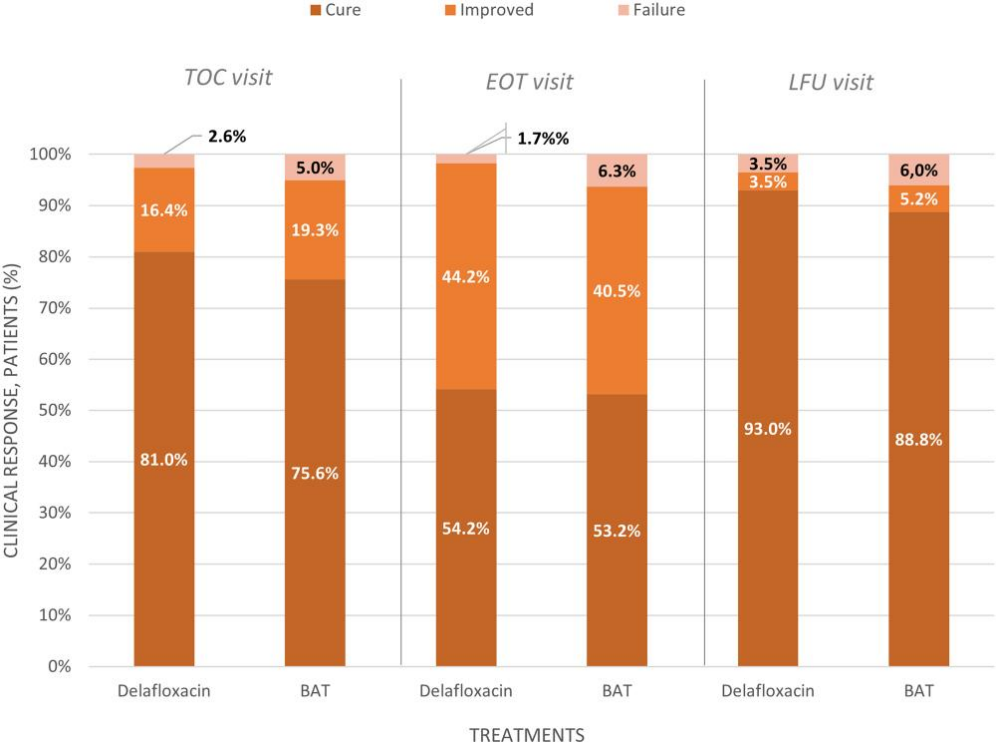
Clinical response at the TOC, EOT, and LFU time points in the CE populations. At TOC visit: CRD = 0.0322 (95% CI, −0.0325 to 0.0969). Noninferiority: met. At EOT visit: CRD = 0.0424 (95% CI, −0.0193 to 0.1040). Noninferiority: met. At LFU visit: CRD = 0.0359 (95% CI, −0.0330 to 0.1049). Noninferiority: met. Confidence intervals were calculated using the Miettinen and Nurminen methods. If the LL of the 2-sided 95% CI was >−0.10, it was concluded that delafloxacin was noninferior to the reference treatment arm for treating patients with cardiothoracic or abdominal SSI. If the LL of the 2-sided 95% CI was >0, then delafloxacin was declared superior to the reference treatment arm for treating patients with cardiothoracic or abdominal SSI. Abbreviations: BAT, best available therapy; CE, clinically evaluable; CRD, common risk denominator; EOT, end of treatment; LFU, last follow-up; SSI, surgical site infection; TOC, test of cure.

In the CE population, more patients achieved clinical success in the delafloxacin group (98.3%) than in the BAT group (93.7%; common risk difference, 0.0424; 95% CI, −0.0193 to 0.1040). Similar distributions between “cure” and “improved” responses were observed also at the EOT visit between the 2 treatment groups (Figure 3).

Delafloxacin was noninferior to BAT for clinical success response at the LFU visit in both the CE and ITT populations (96.5% vs 94.0% and 91.8% vs 87.9%, respectively). “Cure” was the most frequent clinical response in the delafloxacin and BAT group (93% vs 88.8% in the CE population, 88.8% vs 83.3% in the ITT population). No statistically significant superiority was demonstrated in the ITT population (*P* = .3126).

Clinical success achieved at TOC by the 2 treatment arms was sustained through the study period (delafloxacin vs BAT: 95.6% vs 93.1% in the CE population; *P* = .4097; 91% vs 87.1% in the ITT population; *P* = .3046).

### Duration of Treatment, LOS, IRLOS, and Eligibility to Switch to Oral Treatment

In the ITT population, delafloxacin- and BAT-treated patients achieved comparable eligibility to discharge (89% vs 87%) and eligibility to switch to oral treatment (96% vs 98%). Eligibility to switch to oral formulation (48.6 vs 56.8 hours) slightly favored delafloxacin, with time to eligibility to discharge being comparable (132.7 vs 137.6 hours) between the 2 treatment arms.

Delafloxacin treatment offered a shorter, even if not statistically significant, length of stay (LOS) than BAT (mean, 179.4 hours vs 195.4 hours, respectively; *P* = .5604).

Overall treatment duration was shorter for BAT (6.7 days vs 7.7 days), probably due to discontinued visits after discharge of delafloxacin patients switching to oral treatment.

### Microbiological Response

In the MITT population (n = 207), a total of 386 causative pathogens were identified out of 488 isolates collected. In the ME population, 184 patients were selected for the analysis of clinical response at TOC by baseline common pathogen (BCP; ie, ≥5 patients). MRSA-related infection was detected in 4 patients, all randomized to treatment with BAT. At TOC visit, delafloxacin was able to eradicate (either documented or presumed) the infection in 89.5% (94/105) of subjects compared with 79.4% (81/102) of the BAT group in the MITT population (Table 4). The same trend was achieved at TOC in the ME population (91.2% with delafloxacin compared with 78.5% with BAT) and at EOT in the MITT and ME populations.

**Table 4.** Microbiological Response by Type of Infection at TOC Visit—MITT Population

Infection Type	Result	Delafloxacin, No. (%)	BAT, No. (%)
Monomicrobial gram-negative	Eradicated	22 (84.6)	22 (78.6)
	Persisted	4 (15.4)	6 (21.4)
Monomicrobial gram-positive	Eradicated	27 (100)	20 (90.9)
	Persisted	0 (0)	2 (9.1)
Polymicrobial gram-negative	Eradicated	15 (100)	5 (55.6)
	Persisted	0 (0)	4 (44.4)
Polymicrobial gram-positive	Eradicated	2 (100)	4 (100)
	Persisted	0 (0)	0 (0)
Mixed infection	Eradicated	28 (80)	30 (76.9)
	Persisted	7 (20)	9 (23.1)

Abbreviations: MITT, modified intention-to-treat; TOC, test of cure.

Moreover, delafloxacin showed a higher eradication rate compared with BAT for polymicrobial gram-negative infection (100% vs 55.6%, respectively) and a more favorable rate for mixed infection (80.7% vs 75.7%, respectively) at the TOC visit in the ME population (Table 5).

**Table 5.** Clinical Success at TOC in ME Population by Baseline Common Pathogen

Pathogen	Delafloxacin (n = 105), No. (%)	BAT (n = 102), No. (%)
*Escherichia coli*	30/31 (96.7)	38/41 (92.6)
*Enterococcus faecalis*	28/30 (93.3)	30/34 (88.2)
*Enterococcus faecium*	14/14 (100)	12/13 (92.3)
*Pseudomonas aeruginosa*	14/14 (100)	6/9 (66.7)
*Staphylococcus aureus*	7/7 (100)	9/9 (100)
*Morganella morganii*	10/10 (100)	6/6 (100)
*Klebsiella pneumoniae*	8/8 (100)	6/6 (100)
*Enterobacter cloacae*	6/7 (85.7)	5/8 (62.5)
*Proteus mirabilis*	6/6 (100)	5/5 (100)
*Bacteroides fragilis*	4/4 (100)	5/7 (71.4)
*Acinetobacter baumannii*	4/5 (80)	3/3 (100)
*Klebsiella oxytoca*	4/4 (100)	1/1 (100)
*Bacteroides vulgatus*	3/3 (100)	2/2 (100)
*Citrobacter freundii*	1/1 (100)	4/4 (100)

Abbreviations: ME, microbiologically evaluable; TOC, test of cure.

No superinfections were detected during the study, while new infections were observed in both arms at the EOT visit: *E. faecium* was isolated in 1 patient treated with delafloxacin, and a total of 13 different causative pathogens were isolated in 5 patients in the BAT group, for a total of 6 patients in the ITT population (5 patients in the MITT population) reporting clinical failure of antimicrobial therapy.

Superiority of delafloxacin was reached for *Escherichia coli* and *Staphylococcus aureus* at the TOC visit in the ME population (Table 5). At the same time point and in the same population, the noninferiority of delafloxacin was confirmed for other key pathogens such as *Enterococcus faecalis* and *Enterobacter cloacae*.

In patients with superficial infection, delafloxacin was superior to BAT, with a positive outcome in 58/61 vs 46/57, respectively, in the MITT population at TOC (difference, 0.14; 95% CI, 0.02–0.27). The same results were obtained in the ME analysis set and at the EOT visit in both the MITT and ME analysis sets. In patients with deep infection, delafloxacin collected 36 responses out of a total of 44 patients; in the BAT group the responses were 35 out of 45 patients at TOC (MITT population).

### Safety Results

Overall, 58 patients (21.8%) experienced at least 1 TEAE during the study, 32 (23.88%) on delafloxacin and 26 (19.7%) on BAT. The most frequently reported TEAEs corresponded to the standard of care gastrointestinal disorders (5.26% of patients overall), with diarrhea being the most frequently reported (with 3 patients each in the delafloxacin and BAT group) (Table 6).

**Table 6.** Overview of Serious TEAEs in the Safety Population

Type of TEAE	Delafloxacin (n = 134),^a^ No. (%)	BAT (n = 132),^a^ No. (%)	Overall (n = 266),^a^ No. (%)
Overall	13/9 (6.7)	15/14 (10.6)	28/23 (8.6)
Mild	1/1 (0.7)	1/1 (0.8)	2/2 (0.7)
Moderate	4/3 (2.2)	7/6 (4.5)	11/9 (3.4)
Severe	8/5 (3.7)	7/7 (5.3)	15/12 (4.5)
Not related	10/7 (5.2)	14/33 (9.8)	24/20 (7.5)
Possibly related	1/1 (0.7)	0	1/1 (0.4)
Probably related	1/1 (0.7)	0	1/1 (0.4)
Nonassessable	0	1/1 (0.8)	1/1 (0.4)
Unlikely related	1/1 (0.7)	0	1/1 (0.4)
Fatal	3/2 (1.5)	1/1 (0.8)	4/3 (1.1)
Not recovered/not resolved	1/1 (0.7)	0	1/1 (0.4)
Recovered/resolved	9/7 (5.2)	11/11 (8.3)	20/18 (6.8)
Recovered/resolved with Sequelae	0	1/1 (0.8)	1/1 (0.4)
Recovering/resolving	0	2/1 (0.8)	2/1 (0.4)

Abbreviations: BAT, best available therapy; TEAE, treatment-emergent adverse event.

^a^Data are reported as number of events/number of subjects experiencing events (percentages on safety population).

Other TEAEs occurring in at least 3 patients across all treatment groups were hypokalemia (4 patients), pyrexia (3 patients), and pulmonary embolism (3 patients). A slightly higher rate of adverse drug reactions (ADRs) was observed in the delafloxacin treatment arm (5.97% vs 2.3%), with 1 patient with serious ADRs per treatment group (0.75% vs 0.76%).

Twenty-eight serious TEAEs were recorded in 23 patients (9 patients in the delafloxacin group vs 14 patients in the BAT group). Three serious ADRs occurred in 2 patients during the study (2 in the delafloxacin group and 1 in the BAT group), namely *Clostridioides difficile* colitis and septic shock, and 1 death of unknown cause with a drug relationship was reported as nonassessable.

Three patients died during the study: 2 non-treatment-related fatal events occurred in the delafloxacin group and 1 nonassessable death in the BAT group (tigecycline; a case of death of unknown cause).

## DISCUSSION

Acute bacterial skin and skin structure infections represent a frequent reason for hospital admission and a common cause of morbidity in the community [6]. ABSSSIs include cellulitis, abscesses, and wound infections, the last of which may include either trauma or surgical site infections.

While gram-positive pathogens are most common, gram-negative pathogens also play a role in polymicrobial infections, which are especially prevalent in patients with comorbidities and those who have failed prior antibacterial treatment [1, 24].

In the present study, during the microbiological assessment of SSI's causal pathogens, *E. coli* and *P. aeruginosa* accounted for 29.3% and 9.0%, respectively, of baseline common isolated pathogens. *P. aeruginosa* is associated with >300 000 deaths annually [25].

“Antimicrobial Susceptibility Pattern of Bacterial Isolates From SSIs in a Tertiary Care Hospital” showed that *E. coli* was maximally sensitivity to imipenem, piperacillin-tazobactam, and amikacin, least sensitive to ampicillin/sulbactam, ciprofloxacin, and cotrimoxazole, and resistant to cefotaxime and ceftazidime [26]. *P. aeruginosa* has the ability to easily acquire antibiotic resistance determinants and increase resistance to the most commonly used antibiotics such as carbapenem, cefepime, and ceftriaxone [26]. The ability of *P. aeruginosa* to resist the effect of commonly used antibiotics has been escalating exponentially over the years [26]. The combination of chromosomal mutations and the increasing prevalence of transferable determinants of resistance lead to the complex resistance profile of *P. aeruginosa* and its presence on the World Health Organization’s priority list for research and development of new antibiotics [26, 27]. New therapeutic options for the treatment of ABSSSI have become available and offer advantages such as MRSA coverage and the possibility of outpatient treatment (eg, IV to oral switch and/or infrequent administration) [6].

Delafloxacin is the latest addition to the fluoroquinolones in the antibiotic armamentarium, approved by the Food and Drug Administration (FDA) for the oral or intravenous treatment of ABSSSI caused by susceptible bacteria, with a broad-spectrum activity that allows for potential use in infections caused by gram-positive pathogens, including methicillin-resistant *Staphylococcus aureus* (MRSA), and many gram-negative pathogens, including ESBL-producing *Enterobacterales* and *P. aeruginosa,* and anaerobes, without the need for combination therapy, which may reduce antibiotic polypharmacy and its related adverse events and administration time [5, 6, 8, 28].

The DRESS study aimed to expand the utility of delafloxacin in surgical site infections (SSIs), one of the most challenging ABSSSIs to treat in the hospital setting. In this phase IIIb study, delafloxacin was compared with 4 well-established antibiotic standard-of-care therapies, vancomycin/linezolid for cardiothoracic SSI and tigecycline/piperacillin-tazobactam for abdominal SSI.

Data from the present study confirmed delafloxacin’s wide antimicrobial spectrum activity in difficult-to-treat ABSSSIs after cardiothoracic and abdominal surgery, including *E. coli, P. aeruginosa,* and anaerobic pathogens.

There was a higher degree of favorable response (microbiological eradication or presumed eradication) at TOC for delafloxacin-treated patients when compared with BAT. The delafloxacin and BAT groups reported a comparable profile in terms of clinical success, encompassing the clinical response “cure” (resolution of all signs and symptoms) or “improved” (resolution of at least 2 signs or symptoms, allowing antibiotic therapy conclusion). Phase III studies have demonstrated the noninferiority of delafloxacin compared with vancomycin, linezolid, tigecycline, and the combination of vancomycin and aztreonam in the treatment of ABSSSI [6].

Our data confirm findings from a previous multicenter, randomized, double-blind trial of 850 adults with ABSSSI comparing delafloxacin 300 mg IV every 12 hours for 3 days with a switch to 450 mg oral delafloxacin with vancomycin 15 mg/kg IV with aztreonam for 5–14 days. The primary end point was objective response at 48–72 hours. In ABSSSI patients, IV/oral delafloxacin monotherapy was noninferior to IV vancomycin + aztreonam combination therapy for both objective response (83.7% in the delafloxacin arm and 80.6% in the comparator arm) and investigator-assessed response at follow-up and late follow-up (87.2% vs 84.4% and 83.5% vs 82.2%, respectively) [24].

In a double-blind, phase 2 trial of delafloxacin 300 mg compared with 600 mg of linezolid or 15 mg/kg of vancomycin, each administered IV daily for 5–14 days, cure rates were significantly greater with delafloxacin vs vancomycin and similar vs linezolid [29].

The present study has some limitations. Due to the COVID-19 pandemic, which began just 5 months after the first patient enrolled in the study, study execution was challenging. After the first year of patient recruitment, the study counted only 7.8% of patients with cardiothoracic/related leg SSI and 3% of patients coming from Western countries (UK, Italy, Spain, and Austria), with the study population almost entirely enrolled in Eastern European countries and most of the patients affected by abdominal SSI. The epidemiology of the whole European microbiologic panel of SSI pathogens was missed, with a significant imbalance in favor of the Eastern countries. For these reasons, study recruitment was prematurely stopped on September 15, 2020, with a total of 266 patients randomized and treated. The early closure of the study particularly limited the population expected to be randomized to delafloxacin vs linezolid or vancomycin. The COVID-19 pandemic, mainly impacting elective surgeries, caused considerable difficulty in recruiting participants especially within the cardiothoracic SSI group. Even though the projected sample size was not reached, the number of treated patients was sufficient to achieve noninferiority on the primary end point, clinical success rate, with numerical superiority in the delafloxacin arm vs the BAT arm.

The limited number of cardiothoracic/leg SSI patients did not allow for a direct comparison with linezolid, the only comparator with an IV/OS formulation present in the study. Nonetheless, the availability of the oral form of delafloxacin translated to a significantly higher rate of actual discharge before the EOT visit vs the BAT group (68.7% vs 53.8%; *P* = .0125). Early hospital discharge, with assignment of oral treatment to take at home, is likely the reason for the longer duration of treatment recorded in the delafloxacin group vs BAT (7.7 vs 6.7 days), as delafloxacin patients undergo discontinued visits up to EOT compared with the daily visits of hospitalized patients.

The lack of MRSA patients in the delafloxacin-treated population limits the consideration that delafloxacin is noninferior to BAT as an antimicrobial therapy against infections sustained by resistant pathogens. However, in a pooled analysis of 2 multicenter, randomized, double-blind trials of 1510 adults with gram-positive MRSA and gram-negative ABSSSI, IV/oral delafloxacin fixed-dose monotherapy was noninferior to IV vancomycin/aztreonam combination therapy in the eradication of MRSA, with an objective response defined as a ≥20% reduction of lesion spread in the erythema area at the primary infection site at 48 to 72 hours (±2 hours), at 98.1% vs 98.0%, respectively [7]. The efficacy and safety of delafloxacin in the treatment of MRSA ABSSSI were also evaluated in a systematic review and meta-analysis of 4 randomized controlled trials, where delafloxacin exhibited a clinical cure rate and microbiological eradication (documented and presumed) rate similar to the vancomycin and aztreonam, tigecycline, linezolid, and vancomycin comparators in the treatment of ABSSSI and MRSA-associated ABSSSIs [30].

In conclusion, this study supports the noninferiority of delafloxacin monotherapy compared with 4 different available SSI treatments and its superiority compared with BAT for the treatment of both superficial infections and *Escherichia coli/Staphylococcus aureus* infections, offering the advantage of IV/oral formulations and covering the need for an effective empiric treatment against the wide spectrum of pathogens involved in this difficult-to-treat ABSSSI.

The activity against mixed gram-positive and gram-negative infections makes delafloxacin a very attractive option for the treatment of ABSSSI in patients with multiple comorbidities who are at risk of developing polymicrobial infections. Moreover, compared with tigecycline, delafloxacin presents the potential for oral switch, allowing for early patient discharge, and activity against *P. aeruginosa* that can be associated with ABSSSI in selected populations (eg, patients with diabetes, burn wound infections).
